# Multimodal treatments for advanced prostate cancer

**DOI:** 10.18632/oncotarget.26525

**Published:** 2019-01-08

**Authors:** Izak Faiena, Isaac Y. Kim, Thomas L. Jang

**Affiliations:** Thomas L. Jang: Section of Urologic Oncology, Division of Urology, Rutgers Cancer Institute of New Jersey, Rutgers University, New Brunswick, NJ, USA

**Keywords:** prostate cancer, radiation, surgery, survival, combined modality therapy

In 2018, an estimated 164,000 men in the United States will be newly diagnosed with prostate cancer. Among these men, at least 10% will present with locally or regionally advanced disease. In contrast to men with clinically localized prostate cancer, where 5-year relative survival rates approach 100%, men with locally advanced (T3-T4 N0 M0) or regionally advanced (T3-T4 N1 M0) prostate cancer are at high risk of death from their disease [1].

Though the optimal management of these men remains undetermined and widely debated, clinical practice guidelines on prostate cancer—from the National Comprehensive Cancer Network (NCCN) and the European Association of Urology-European Society for Radiotherapy and Oncology-International Society of Geriatric Oncology (EAU-ESTRO-SIOG)—generally support multimodal treatment approaches, which include radical prostatectomy followed by adjuvant radiotherapy or radiotherapy plus androgen deprivation therapy (ADT). Randomized prospective trials have demonstrated combination therapies to improve survival and cancer outcomes when compared to monotherapy for men with high-risk prostate cancers [2, 3].

For example, the European Organization for Research and Treatment of Cancer (EORTC) 22863 trial randomized patients with high metastatic risk (90% of patients had clinical T3-T4 disease) and found clear efficacy of radiotherapy with long-term ADT when compared with radiotherapy alone. In this study, 10-year overall survival was 39.8% (95% CI 31.9 - 47.5) in patients receiving radiotherapy alone *versus* 58.1% (95% CI 49.2 - 66.0) in the combined treatment group and 10-year prostate-cancer mortality was 30.4% (95% CI 23.2 - 37.5) and 10.3% (95% CI 5.1 - 15.4) in the radiotherapy and combination therapy groups, respectively [4]. Similarly, the Radiation Therapy Oncology Group (RTOG) 85-31 trial showed that for patients with clinical T3 or N1 M0 disease, ADT applied in the adjuvant setting after definitive radiotherapy when compared to radiotherapy alone was associated with a reduction in disease progression and improvement in overall survival [5].

Several prospective trials evaluating primary radical prostatectomy followed by adjuvant radiotherapy *versus* radical prostatectomy alone have shown the combination group to improve biochemical recurrence-free survival and local control when compared to radical prostatectomy alone. EORTC trial 22911 randomized men to observation or adjuvant radiotherapy after radical prostatectomy. At a median follow-up of 10.6 years, men who received adjuvant radiotherapy experienced improved biochemical progression-free survival compared with the observation group (39.4% *vs* 61.8%, respectively, *p* < 0.0001) [6]. Southwest Oncology Group (SWOG) 8794 randomized men with stage pT3N0M0 who had undergone radical prostatectomy to receive adjuvant radiotherapy or usual care plus observation and found that adjuvant radiotherapy significantly reduced the risk of PSA relapse (median PSA-relapse free survival, 10.3 years for radiotherapy *vs* 3.1 years for observation, *p* < 0.001) and disease recurrence (median recurrence-free survival, 13.8 years for radiotherapy *vs* 9.9 years for observation, *p* = 0.001) [7].

Based on level 1 evidence, it is clear that multimodal treatments for these men are associated with superior outcomes when compared to single treatment alone. However, little comparative data exists to compare the two substantially different treatment approaches generally accepted for these men. Recently, we reported results of a comparative effectiveness study of radical prostatectomy with adjuvant radiotherapy *versus* radiotherapy plus ADT for men with advanced prostate cancer [8].

Radical prostatectomy has not conventionally been used for high-risk prostate cancer, as the procedure in this setting can be technically challenging, and carries an increased risk of positive margins, biochemical recurrence of PSA, or distant relapse. As a result, management trends have shifted away from surgical treatment toward higher utilization of radiotherapy plus ADT. The theoretical benefits of radical prostatectomy in first-line treatment are tumor volume reduction and optimal local control, and more accurate staging, risk stratification, and pathologic assessment of tumor characteristics that will better select for men who may benefit from adjuvant treatment.

Using data from the Surveillance, Epidemiology, and End Results (SEER)-Medicare program, we identified 13,856 men 65 years or older from 1992 to 2009 who were diagnosed with locally advanced (T3-T4N0M0) or regionally advanced (T3-T4N1M0) prostate cancer, of which 6.1% received radical prostatectomy with adjuvant radiotherapy and 23.6% received radiotherapy plus ADT. The data were obtained from 17 affiliated cancer registries and represented 28% of the U.S. population. Several key findings are notable. First, non-adherence to the NCCN and EAU-ESTRO clinical practice guidelines was observed, as almost half the entire study cohort received a single intervention and almost 20% received no treatment within six months of diagnosis. Not surprisingly, patient age, comorbid conditions, and cancer stage were associated with treatment received. Second, men who received radical prostatectomy followed by radiotherapy were significantly less likely to die from prostate cancer and had improved overall survival than men who received radiotherapy plus ADT. These findings were independent of primary tumor stage, nodal stage, or Gleason score, although the survival advantage benefited men without lymph node metastases most. The adjusted 10-year prostate cancer-specific survival rates for men with T3a/bN0M0 disease, T3a/bN1M0 disease, and T4N0M0 disease were 88.9%, 75.7%, and 72%, respectively, for those who received radical prostatectomy followed by radiotherapy and 74.2%, 58.6%, and 60.5%, respectively, for those who received radiotherapy plus ADT. Third, men who underwent radical prostatectomy with radiotherapy had higher rates of erectile dysfunction (28% *vs* 20%) and higher rates of urinary incontinence (49% *vs* 19%) in comparison with those who underwent radiotherapy plus ADT [7].

This data suggests that even men with high-risk prostate cancer that is not clinically localized can achieve durable long-term survival when a multimodal treatment strategy is employed. Since the study was observational in design and men were not randomized to treatment approaches, the findings are suggestive and limited by the usual biases of an observational study design using administrative claims. It is important to note also, that since 2009, the final year in which data was captured in this study, significant advances in prostate cancer surgery and radiotherapy approaches have been made to improve outcomes and minimize treatment adverse effects, such as erectile dysfunction and urinary incontinence.

In summary, our study demonstrates that men with locally advanced or regionally advanced prostate cancer who received primary radical prostatectomy with adjuvant radiotherapy had a lower risk of death from their disease and experienced improved overall survival in comparison with those treated with primary radiotherapy plus ADT. The Scandinavian Prostate Cancer Group-15 (SPCG-15) trial, a prospective, randomized phase III trial comparing radical prostatectomy (with or without adjuvant or salvage radiotherapy) with primary radiotherapy plus ADT among patients with locally advanced prostate cancer, will provide clarity on the optimal management of patients with high-risk disease [9]. Until this data is available, future trial design should consider a surgical arm and should focus on optimizing treatment sequences and timing, optimizing intensities of treatment, and integrating more effective systemic therapies with optimal local treatments.
